# Supplementary material for: Electrochemical Properties of an Sn-Doped LATP Ceramic Electrolyte and Its Derived Sandwich-Structured Composite Solid Electrolyte
Source: Nanomaterials (Basel). 2022 Jun 16;12(12):2082. doi: 10.3390/nano12122082 (PMC9228486; doi:10.3390/nano12122082)
Supplement: Supplementary file 1 [file nanomaterials-12-02082-s001.zip › nanomaterials-1747240-supplementary.pdf]

nanomaterials

Supplementary Materials

Electrochemical Properties of an Sn-Doped LATP Ceramic Electrolyte and Its Derived Sandwich-Structured Composite Solid Electrolyte

Aihong Xu [1,2], Ruoming Wang [1,2], Mengqin Yao [1,2], Jianxin Cao [1,2], Mengjun Li [1,2], Chunliang Yang [1,2], Fei Liu [1,2,*] and Jun Ma [1,2,*]

[1] Department of Chemical Engineering, School of Chemistry and Chemical Engineering, Guizhou University, Guiyang 550025, China; 15851959055@163.com (A.X.); w1285459785@126.com (R.W.); mqyao@gzu.edu.cn (M.Y.); jxcao@gzu.edu.cn (J.C.); x1285459785@126.com (M.L.); clyang@gzu.edu.cn (C.Y.)

[2] Guizhou Key Laboratory for Green Chemical and Clean Energy Technology, Guiyang 550025, China

* Correspondence: ce.feiliu@gzu.edu.cn (F.L.); jma3@gzu.edu.cn (J.M.)

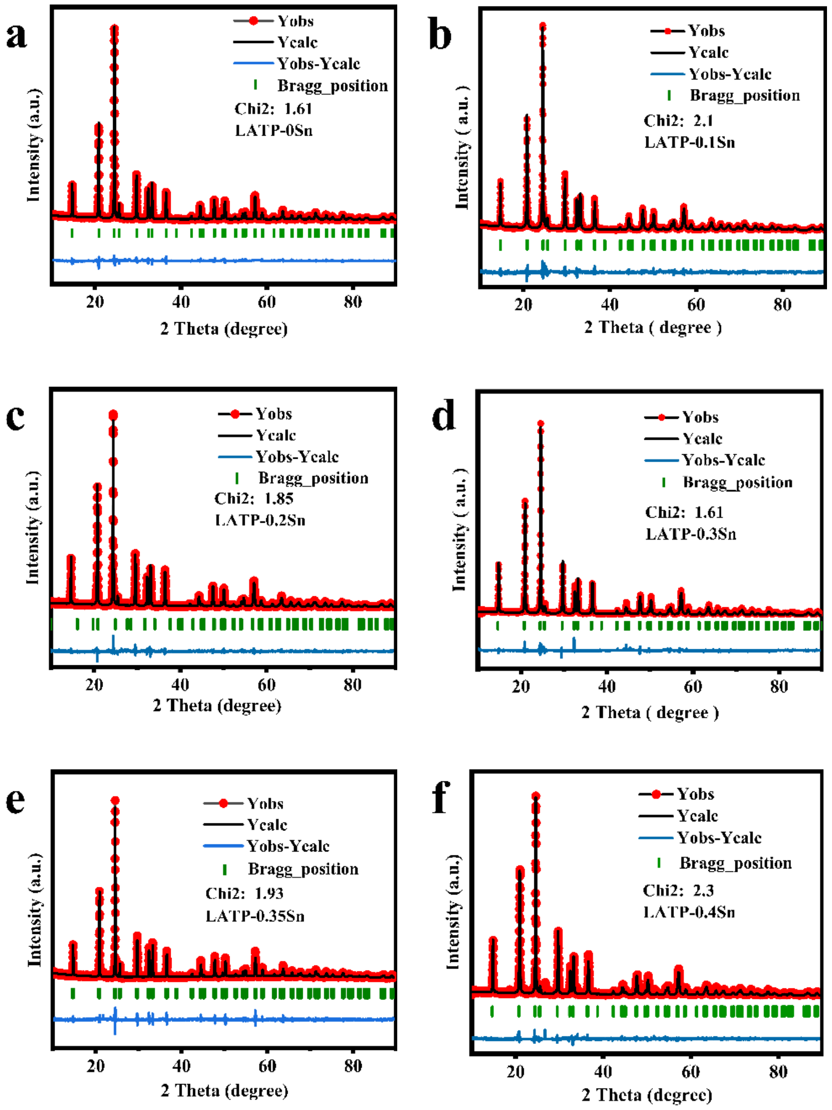

Figure S1. Rietveld refinement of XRD patterns of LATP-xSn (x = 0–0.4) solid electrolytes.

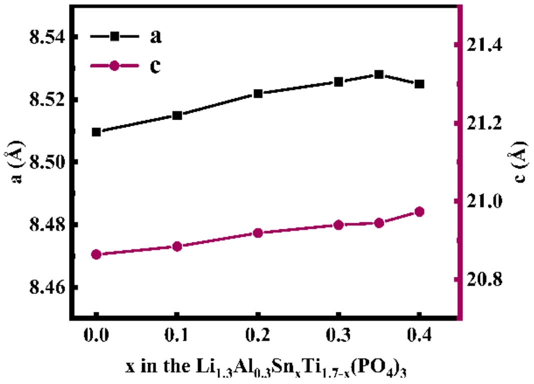

Figure S2. Lattice parameters of LATP-xSn (x = 0–0.4) solid electrolytes.

Table S1. The deviation degrees of Sn^{4+} at P^{5+} site, Al^{3+} site and Ti^{4+} site respectively under different coordination environments.

Ions	Sn^{4+}	P^{5+}	Al^{3+}	Ti^{4+}
Crystal ionic radii with tetrahedral coordination	69 pm	31 pm	/	/
Crystal ionic radii with octahedral coordination	83 pm	/	67.5 pm	74.5 pm
Electronegativity with tetrahedral coordination	1.95	3.003	/	/
Electronegativity with octahedral coordination	1.706	/	1.513	1.730
Deviation degree	/	1.58	0.357	0.128

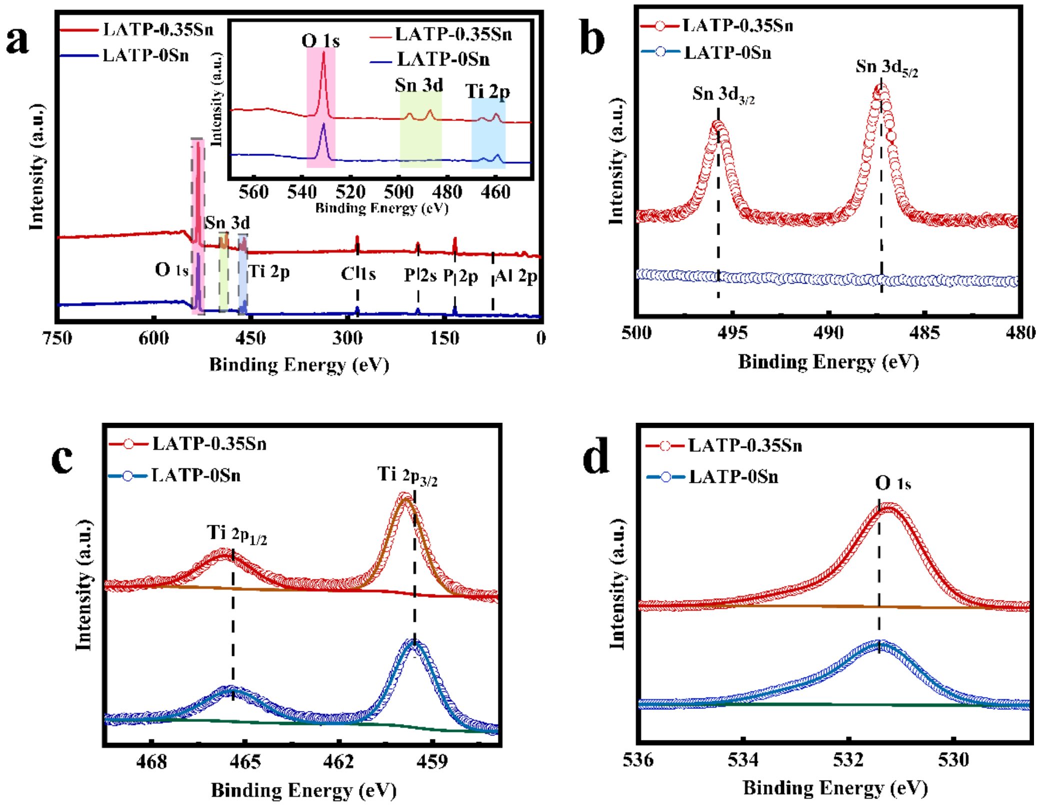

Figure S3. XPS spectra of LATP-0Sn and LATP-0.35Sn solid electrolytes (**a**) survey spectra; (**b**) Sn 3d spectra; (**c**) Ti 2p spectra; (**d**) O 1s spectra.

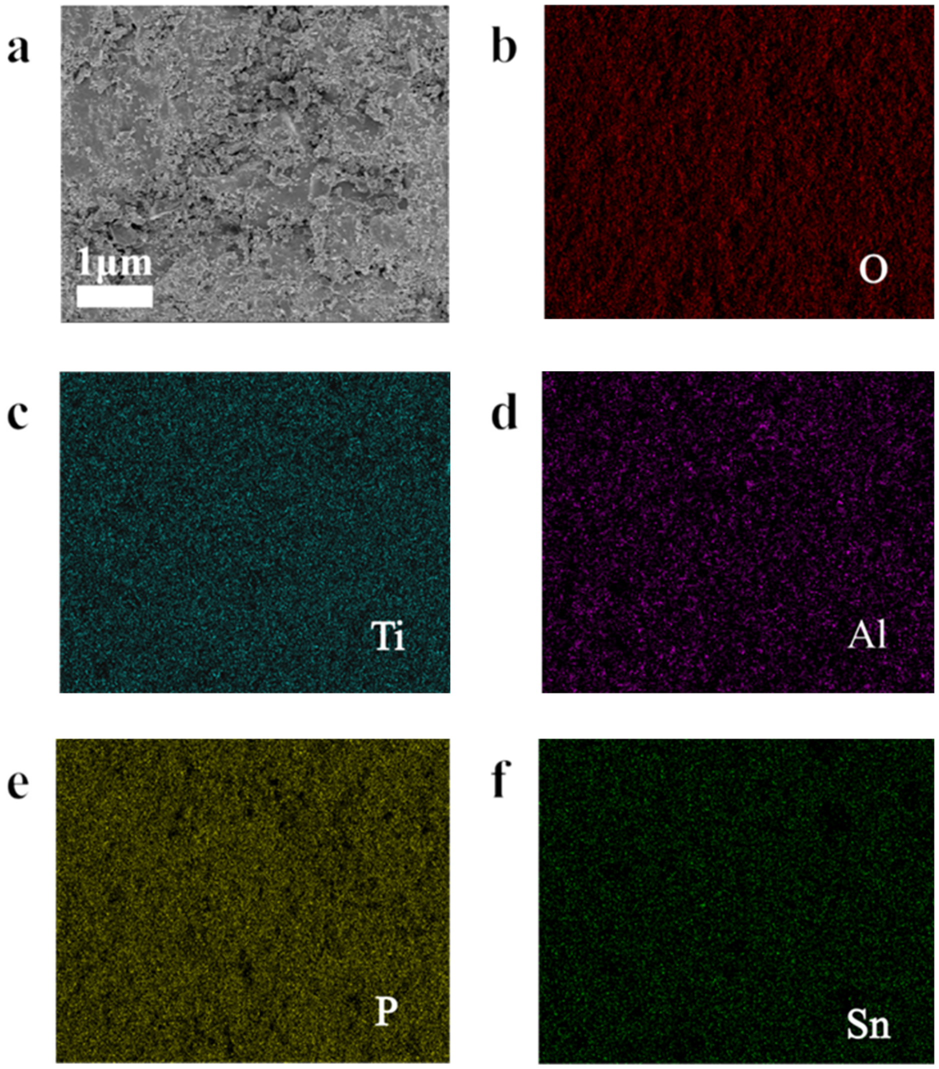

Figure S4. (**a**) SEM image of LATP-0.35Sn solid electrolyte and (**b**–**f**) the corresponding EDS elemental maps.

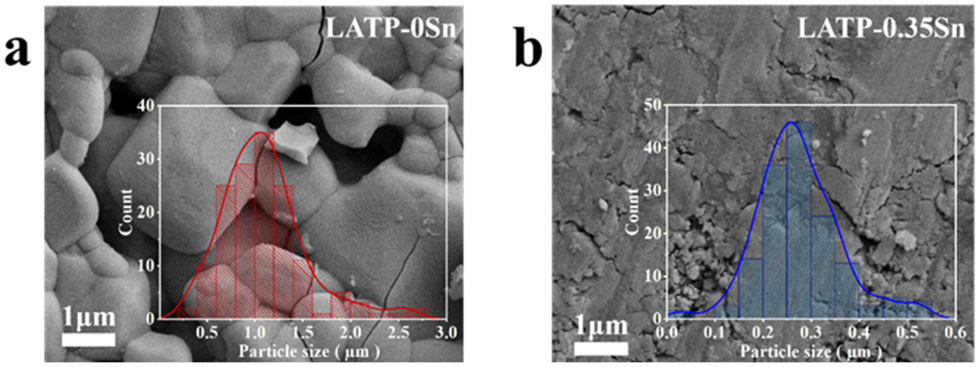

Figure S5. SEM images and grain size distribution curves of (**a**) LATP-0Sn solid electrolyte and (**b**) LATP-0.35Sn solid electrolyte.

Table S2. Performance comparison of LATP-xSn obtained with other typical solid electrolytes.

Composition	Electrolyte-type	Preparation method	Conductivity (mS/cm)	Reference
$Li_7La_3Zr_2O_{12}$	Garnet	solid-state reaction	0.38	58
$Li_{3/8}Sr_{7/16}Hf_{1/4}Ta_{3/4}O_3$	Perovskite	solid-state reaction	0.38	59
$Li_{10.42}Ge_{1.5}P_{1.5}Cl_{0.08}O_{11.92}$	LISICON	melt-casting	0.037	60
Li_3PO_4	LiPON	magnetron sputtering	0.001	61
β-Li_3PS_4	Sulfides	liquid-phase reaction	0.27	62
Li_3OCl	Anti-perovskite	PLD	0.2	63

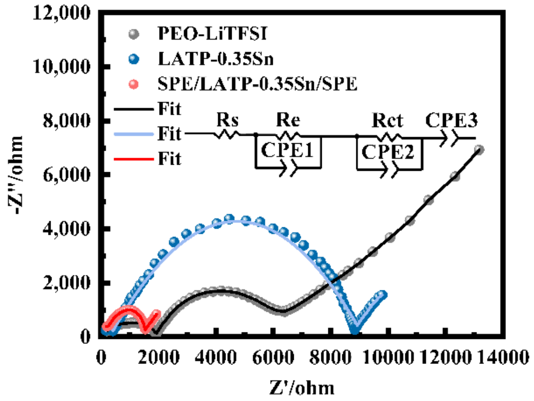

Figure S6. EIS curves of LATP-0.35Sn and SPE/LATP-0.35Sn/SPE solid electrolytes at 25 °C.

Table S3. Electrolyte resistance (Re), charge transfer resistance (Rct), total resistance (Rt) and total ionic conductivities (σt) of PEO-LiTFSI, LATP-0.35Sn and SPE/LATP-0.35Sn/SPE solid electrolytes at 25 °C.

Electrolytes	R_e (Ω)	R_{ct} (Ω)	R_t (Ω)	σ_t (S/cm)
PEO-LiTFSI	1739	4408	6344	1.0×10^{-6}
LATP-0.35Sn	413	8514	8927	1.0×10^{-5}
SPE/LATP-0.35Sn/SPE	239	1344	1583	5.9×10^{-5}

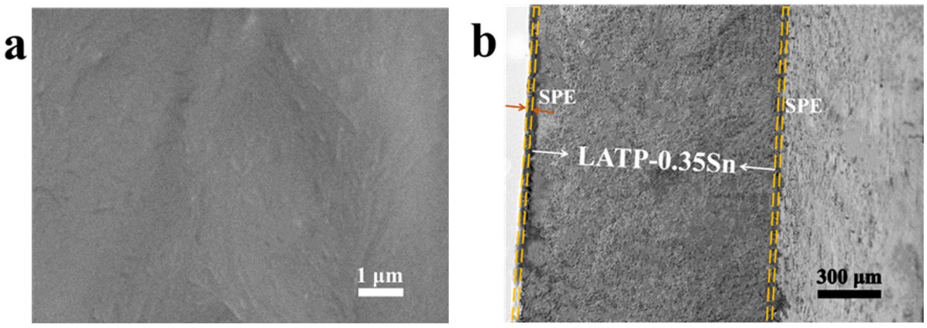

Figure S7. SEM images of SPE/LATP-0.35Sn/SPE solid electrolytes (**a**) surface and (**b**) cross-section.
